# Adverse associations of car time with markers of cardio-metabolic risk

**DOI:** 10.1016/j.ypmed.2015.11.029

**Published:** 2016-02

**Authors:** Takemi Sugiyama, Katrien Wijndaele, Mohammad Javad Koohsari, Stephanie K. Tanamas, David W. Dunstan, Neville Owen

**Affiliations:** a Centre for Design Innovation, Faculty of Health Arts & Design, Swinburne University of Technology, Melbourne, VIC, Australia; b School of Population Health, University of South Australia, Adelaide, SA, Australia; c Baker IDI Heart and Diabetes Institute, Melbourne, VIC, Australia; d Melbourne School of Population and Global Health, The University of Melbourne, Melbourne, VIC, Australia; e MRC Epidemiology Unit, University of Cambridge, Cambridge, United Kingdom; f School of Exercise and Nutrition Sciences, Deakin University, Burwood, VIC, Australia; g School of Population Health, The University of QLD, Brisbane, QLD, Australia; h Department of Epidemiology and Preventive Medicine, Monash University, Melbourne, VIC, Australia; i School of Sport Science, Exercise and Health The University of Western Australia, Perth, WA, Australia; j Central Clinical School, Monash University, Melbourne, VIC, Australia; k Mary MacKillop Institute of Health Research, Australian Catholic University, Melbourne, VIC, Australia

**Keywords:** Sedentary behavior, Motorized transport, Automobile, Adiposity

## Abstract

**Objective:**

To examine associations of time spent sitting in cars with markers of cardio-metabolic risk in Australian adults.

**Method:**

Data were from 2800 participants (age range: 34–65) in the 2011–12 Australian Diabetes, Obesity and Lifestyle Study. Self-reported time spent in cars was categorized into four groups: ≤ 15 min/day; > 15 to ≤ 30 min/day; > 30 to ≤ 60 min/day; and > 60 min/day. Markers of cardio-metabolic risk were body mass index (BMI), waist circumference, systolic and diastolic blood pressure, triglycerides, HDL (high-density lipoprotein)-cholesterol, fasting plasma glucose, 2-h plasma glucose, a clustered cardio-metabolic risk score, and having the metabolic syndrome or not. Multilevel linear and logistic regression analyses examined associations of car time with each cardio-metabolic risk outcome, adjusting for socio-demographic and behavioral variables and medication use for blood pressure and cholesterol/triglycerides.

**Results:**

Compared to spending 15 min/day or less in cars, spending more than 1 h/day in cars was significantly associated with higher BMI, waist circumference, fasting plasma glucose, and clustered cardio-metabolic risk, after adjusting for socio-demographic attributes and potentially relevant behaviors including leisure-time physical activity and dietary intake. Gender interactions showed car time to be associated with higher BMI in men only.

**Conclusions:**

Prolonged time spent sitting in cars, in particular over 1 h/day, was associated with higher total and central adiposity and a more-adverse cardio-metabolic risk profile. Further studies, ideally using objective measures of sitting time in cars and prospective designs, are needed to confirm the impact of car use on cardio-metabolic disease risk.

## Highlights

• Time spent in cars was adversely associated with markers of cardio-metabolic risk.
• Spending more than 1 h per day in cars may be particularly detrimental.
• Future research with objective measures of car time and prospective design is needed.

## Background

High volumes of daily sitting time, which are now common in many countries (Matthews et al., 2008, Ng and Popkin, 2012) are associated with an increased risk of cardiovascular and other chronic diseases (Cooper et al., 2014, Healy et al., 2008, Owen et al., 2010, Thorp et al., 2010, Wijndaele et al., 2014). Of the specific sedentary behaviors, TV viewing time has been examined extensively and shown to be associated with cardiovascular mortality (Dunstan et al., 2010, Matthews et al., 2012) and markers of cardio-metabolic risk (Inoue et al., 2012, Thorp et al., 2010, Wijndaele et al., 2010). However, potential health effects of other sedentary behaviors, such as sitting in cars, have been less studied. Although sitting defines sedentary behaviors regardless of where they take place, driving can involve slightly higher energy expenditure (2.0 METs) than other sedentary behaviors such as TV viewing (sitting quietly, 1.0 METs) and sitting at work (1.5 METs) (Ainsworth et al., 2000). Recent studies have suggested that sitting in different domains may not be similarly associated with mortality and disease outcomes (Basterra-Gortari et al., 2014, Chau et al., 2012, Pinto Pereira et al., 2012). Given that different strategies may be needed to reduce sedentary behavior in different domains (leisure, transport, and occupation), it is important for practitioners and policy makers in relevant sectors to understand how particular domain-specific sedentary behaviors are related to health risk.

The proportion of adults who use a car as the main form of transport to work or for other commuting purposes is high: 86% in the USA and 78% in Australia (Australian Bureau of Statistics, 2012, McKenzie and Rapino, 2011). Even in European countries, which can have more-compact urban environments and more accessible public transport, the majority of trips are made by cars. For example, cars are used for 64% of all instances of travel in the UK, and 53% in Sweden (Transport Analysis, 2012, UK Department of Transport, 2013). Car use is not only high in frequency, but can also be substantial in duration. Australian household travel surveys show that adults spend on average more than 50 min/day in a car (Ironmonger, 2008), with up to 18% of men and 12% of women spending more than 2 h/day (Sugiyama et al., 2012).

Car commuters have been shown to have higher odds of being overweight or obese, compared to non-car commuters (Laverty et al., 2013, Lindstrom, 2008). Frequent car use for commuting and errands has been associated with higher BMI (Pendola and Gen, 2007). A recent review has shown that longer car use (measured in time or in distance) was significantly associated with higher weight status in eight out of 10 studies (McCormack and Virk, 2014). Longitudinal studies have also found frequent and longer car use to be associated with greater weight gain and higher cardiovascular mortality (Sugiyama et al., 2013, Warren et al., 2010). These studies, however, have used predominantly self-reported adiposity measures. One study reported distance between home and work to be associated with a metabolic syndrome risk score derived from objectively-assessed biomarkers (Hoehner et al., 2012). However, such a distance measure focusing on the workplace may not accurately represent time spent sitting in cars.

As discussed above, car use is a common sedentary behavior among adults. Since it has a broad detrimental impact on society including human health, greenhouse gas emission, and congestion, producing specific evidence of how time in car is associated with increased risk of chronic diseases is relevant to help inform policies to promote active travel. We examined associations of time spent sitting in cars with objectively-measured markers of cardio-metabolic risk among Australian adults.

## Methods

### Data source

Data were obtained from participants of the third wave of the Australian Diabetes, Obesity and Lifestyle (AusDiab3) study, which was conducted in 2011–12. AusDiab is a cohort study that was originally established to examine the national prevalence and incidence of diabetes and related risk factors. Detailed methods of recruitment and data collection have been reported elsewhere (Dunstan et al., 2002, Thorp et al., 2010). Briefly, the initial data collection (AusDiab1) was conducted in 1999–2000, using a stratified cluster sampling method. First, six Census Collection District (CCD, a geographic unit defined by the Australian Bureau of Statistics with an average of 225 dwellings each) clusters were selected from each of the seven Australian states and territory. Within each of the 42 study areas, adults aged 25 years and over who had resided at the address for 6 months or longer prior to the survey and those without physical or intellectual disabilities were asked to participate in the survey. The sample for AusDiab 1 (n = 11,247) was a national sample of the adult population aged 25 years and over. Survey questions on car use were first introduced in AusDiab3 (n = 6,186). The response rate for this wave among eligible AusDiab2 participants was 59.8%. Of these, 4,614 participants attended testing sites for biomedical examination (Tanamas et al., 2013). The sample for the current study consisted of those who were 65 years or younger (n = 3,112). Adults older than 65 years were excluded from this analysis on the grounds that cardio-metabolic health of those who were retired (no longer driving to work) may have been influenced by previous driving habits. Of these, participants who did not report car time (n = 86), did not have more than one outcome measure (n = 21), did not answer a general questionnaire for socio-demographic variables (n = 63), were diagnosed with diabetes (n = 138), had a history of cardiovascular disease (angina, heart attack, stroke; n = 51), or were pregnant (n = 5) were excluded. The final sample size was 2,800 (exclusion criteria not mutually exclusive). The study was approved by the Ethics Committee of the International Diabetes Institute and the Alfred Health Human Ethics Committee, and written informed consent was obtained from all participants.

### Outcome measures

Objective markers of cardio-metabolic risk included BMI; waist circumference; systolic and diastolic blood pressure (BP); triglycerides; HDL-cholesterol; fasting plasma glucose; 2-h post-load plasma glucose; clustered cardio-metabolic risk; and having the metabolic syndrome or not. Trained personnel at data collection sites measured participants' height, weight, waist circumference, and resting BP. Fasting serum triglycerides, HDL-cholesterol, and fasting and 2-h plasma glucose levels were measured by enzymatic methods using the Roche Modular (Roche Diagnostics, Indianapolis, IN). A continuous clustered cardio-metabolic risk score was computed using five cardio-metabolic measures: waist circumference; BP (average of systolic and diastolic); triglycerides; HDL-cholesterol; and fasting plasma glucose (Wijndaele et al., 2009). After standardizing all five markers, the risk score was calculated by summing all standardized scores and dividing this sum by five. Gender-specific means and standard deviations from all participants with complete data for each cardio-metabolic variable were used. A higher positive score denotes greater cardio-metabolic risk. Triglycerides and fasting plasma glucose were normalized (natural log) prior to standardization. The standardized score of HDL-cholesterol was multiplied by minus one, to account for its protective cardio-metabolic effect. The presence of the metabolic syndrome was determined based on the 2009 Joint Interim Statement (Alberti et al., 2009).

### Exposure measure

Participants were asked to report the total duration they used a car (as driver or passenger) to get to places such as work, school, shops, or services in the last week. The question was asked in the format used for those in the International Physical Activity Questionnaire (Craig et al., 2003). Participants were categorized into four groups according to daily time spent in cars: ≤ 15 min/day; > 15 to ≤ 30 min/day; > 30 to ≤ 60 min/day; and > 60 min/day.

### Covariates

Socio-demographic variables were collected using an interviewer-administered questionnaire. They included age; gender; education (high school or less, technical/vocational, bachelor's degree or higher); work status (working including students, not working, other); marital status (single, couple); having children in the household (yes, no); and annual household income (less than AU$60 K, AU$60–125 K, AU$125 K or more, no response). Behavioral covariates included self-reported sitting time for work, TV viewing time, leisure-time computer use, leisure-time moderate-to-vigorous physical activity (LTPA), energy intake, and alcohol consumption. Participants reported time spent sitting as part of their work in the last week during weekdays (sitting for work), time spent sitting to watch TV or video/DVD in the last week (TV viewing), and time spent sitting to use a computer, the internet, and electronic games during leisure time in the last week (computer use). For each of them, the average daily sitting time was calculated. LTPA was determined using the Active Australia Survey, a validated and reliable questionnaire, by summing the time spent in moderate physical activity and vigorous physical activity (multiplied by two) in the last week (Armstrong et al., 2000). Daily energy intake and alcohol consumption were determined using a self-administered food-frequency questionnaire, with a reference frame of the last 12 months (Hodge et al., 2000). Daily intakes of each food were calculated using sex-specific standard portion sizes derived from weighed food records and the reported frequencies converted to daily equivalents. From these data, intakes of nutrients, including energy, were calculated using NUTTAB95 food composition data (Lewis et al., 1995). In addition, self-reported medication use for BP and for cholesterol or triglycerides were also included as covariates.

### Statistical analyses

Outcome variables that had a skewed distribution (triglycerides, fasting plasma glucose, and 2-h plasma glucose) were log-transformed to reduce skewness. Multilevel linear and logistic regression analyses examined associations of time spent in cars with each outcome, using CCD clusters as a random intercept. Analyses adjusted for socio-demographic and behavioral variables described above. They also adjusted for medication use for BP (only in models examining systolic BP, diastolic BP, and cardio-metabolic risk) and medication for cholesterol/triglycerides (only in models examining triglycerides, HDL-cholesterol, and cardio-metabolic risk). Results were shown in unstandardized regression coefficients for continuous outcome measures and odd ratios for the metabolic syndrome, according to each car time category with p values for linear trend. In light of previous studies showing differential associations of sedentary behavior with health outcomes between men and women (Thorp et al., 2010, Yates et al., 2012), gender interaction was also examined for each outcome. We also examined the interaction of work status with time in cars, as workers and non-workers are likely to differ in their behavior pattern. When the interaction term approached significance (p ≈ 0.1), stratified analyses were conducted. To examine whether other behavioral variables (sitting, LTPA) attenuate the association of time in cars and the outcomes, we also conducted separate analyses where each behavior is adjusted for separately. Analyses were conducted using STATA version 12 (StataCorp, College Station, TX). Statistical significance was set at p < 0.05.

## Results

Table 1 shows the characteristics of the study sample (n = 2800). Participants' average age was 54 years (range: 34–65 years), and more than two thirds were working. The mean daily duration in cars was 49 min, which is similar to the average daily duration of car use derived from household travel surveys in Australia (Ironmonger, 2008). Time spent in cars was weakly correlated with other behavioral variables: correlation coefficients were 0.06 (p = 0.005) with sitting for work, − 0.04 (p = 0.04) with TV viewing, − 0.04 (p = 0.02) with leisure-time computer use, and 0.02 (p = 0.3) with LTPA.

Table 2 shows the results of the regression analyses for each outcome measure. Compared to participants using a car for 15 min/day or less, those who spent more than 1 h/day in cars were more likely to have significantly higher BMI, waist circumference, fasting plasma glucose, and clustered cardio-metabolic risk. Using a car between 30 and 60 min/day was also associated with higher clustered cardio-metabolic risk. Car time was not significantly associated with BP, triglycerides, HDL-cholesterol, 2-h plasma glucose, and the metabolic syndrome. In addition to the full model, we further examined to what extent the associations between car time and the outcomes were attenuated by other behaviors. It was found that sitting for work and LTPA slightly attenuated the associations, but TV viewing and leisure-time computer use strengthened the association (Supplementary Tables 1–5). However, the overall results (car time being associated with BMI, waist circumference, fasting plasma glucose, and clustered cardio-metabolic risk) remained similar after adjusting for each behavioral covariate.

Interaction analyses found that the interaction between gender and time spent in cars on BMI approached significance (p = 0.11). Table 3 shows the results of the regression analyses for BMI, stratified by gender. A significant association between time in cars and BMI was observed for men but not for women. Interaction of work status with time in cars was not significant for any of the outcome variables.

## Discussion

In our sample of Australian adults, we observed that those who spent longer time in cars showed higher levels of general and central adiposity and a poorer overall cardio-metabolic risk profile, after adjusting for several potential confounders. Relative to participants who spent 15 min/day or less in cars, those who spent more than 1 h/day (about a quarter of the sample) were likely to have a 0.8 greater BMI (equivalent to 2.3 kg for a person with a height of 1.7 m), and 1.5 cm greater waist circumference. Previous studies have shown the associations of car use with self-reported adiposity measures (Ding et al., 2014, Frank et al., 2004, Laverty et al., 2013, Lindstrom, 2008, Sugiyama et al., 2013). Another study showed that living far from work was detrimentally associated with objectively-assessed BMI, waist circumference, blood pressure, and continuous metabolic syndrome score (Hoehner et al., 2012). Our study adds further evidence supporting the unfavorable associations of prolonged car use with objectively-measured markers of cardio-metabolic risk, particularly among those who used a car more than 1 h/day.

Our findings on car time are broadly comparable with previous studies using objectively-measured total sedentary time. They showed mostly consistent significant associations with adiposity and with cardio-metabolic risk, and consistent non-significant associations with blood pressure (Cooper et al., 2014, Healy et al., 2008, Wijndaele et al., 2014). However, the current study differed from these previous studies in associations with triglycerides and fasting plasma glucose. Compared to leisure-time sitting, driving a car can involves some modest bodily movement (Ainsworth et al., 2000), and occurs in a setting that can be stressful (Ding et al., 2014, Rissel et al., 2014). Such differences may provide an explanation for the variations between this study and those using objectively-measured overall sedentary time. It should be noted that car use as a passenger was also included in this study. However, in Australia, a large majority of car use is done by unaccompanied drivers (Australian Bureau of Statistics, 2012).

We found that time spent sitting in cars was only weakly correlated with other sedentary behaviors and leisure-time physical activity. Additional analyses in which these behavioral covariates were adjusted for individually also showed that the associations of time in cars with the outcomes were not markedly influenced by other behaviors (Supplementary Tables). The results suggest that car use is mostly unrelated to other behaviors measured in the study (e.g., sitting for work, TV viewing), and independently associated with markers of cardio-metabolic risk. Further research examining the relative contributions of domain-specific sitting (at home, in car, at work) to health outcomes is warranted to develop more effective sitting interventions to enhance population health.

Interaction analyses showed that time in cars was associated with BMI for men but not for women. It was found that men who spent more than 30 min/day in cars had a significantly higher BMI, compared to those who spent 15 min/day or less in cars. Previously, studies reported stronger associations between sedentary time and adiposity measures for women (Thorp et al., 2010, Yates et al., 2012). Studies have shown prolonged bouts of sitting to be associated with cardio-metabolic risk (Carson et al., 2014, Healy et al., 2011). There may be gender differences in the duration of bouts in car use, which may partially account for the differential association. Further studies identifying the bout of driving would be of interest to understand whether it is related to other measures of cardio-metabolic health.

Strengths of the study include the use of objectively-measured markers of cardio-metabolic risk, a large sample obtained from diverse locations (urban, suburban, and regional) throughout Australia, and adjustment by behavioral (including dietary intake) variables and medication use. However, there are limitations to be recognized. First, the cross-sectional nature of our study hinders any inference on causality. It is possible that participants with high adiposity may have spent a longer time in cars as they find the use of public transport inconvenient. It is also possible that they have chosen to live in a car-oriented neighborhood. Longitudinal studies, considering the reason for moving to the neighborhood, are needed to confirm our findings. Second, this study used a self-report measure for time spent in cars, which may involve recall error. To date, however, no studies have directly measured time spent sitting in cars. In the literature, duration was estimated from the distance between the origin and destination reported in travel surveys (Frank et al., 2004), or the distance between home and work was used as an exposure measure (Hoehner et al., 2012). Given that car use is a common sedentary behavior among adults, objective methods to accurately identify how long people sit in a car need to be developed and employed in future research. Global positioning systems, which are now widely available in mobile phones and have begun to be used for health research (Kerr et al., 2011), may be employed for this purpose. Third, our findings may be influenced by residual confounding of unmeasured factors, such as sitting in other types of transportation (e.g., train, bus), stress, and exposure to particulate matter during car use (Chertok et al., 2004).

In conclusion, this study adds further evidence on the likely adverse cardio-metabolic health consequences of prolonged time in cars, in particular for a quarter of the sample who spent over 1 h per day in cars. Transport sectors have been trying to promote active travel mainly to reduce congestion, air pollution, and the proliferation of automobile-related infrastructure. Such efforts can be further supported by producing a compelling body of evidence on the adverse health impact of prolonged time spent in cars. Research on the deleterious health impacts of car use can contribute to more comprehensive evidence base to underpin advocacy of active transport options. Collaborative research between the health (including health economics), transport, and planning sectors has considerable potential to promote active travel further and to broaden the base for cardio-metabolic disease prevention initiatives.

## Conflict of interest statement

The authors declare that there are no conflicts of interests.

## Transparency document

Transparency document.

## Figures and Tables

**Table 1:** Characteristics of the sample, the 2011–12 AusDiab study (n = 2800).

	Mean (SD), median (interquartile range) or %
Age	54.4 (7.3)
Gender, %women	57.0
Education	
%High school or less	29.2
%Technical/vocation	43.1
%Bachelor's degree or more	27.7
Work status	
%Working (including students)	70.0
%Not working	27.4
%Other	2.6
Marital status, %couple	82.1
Child or children in the household, %yes	42.6
Annual household income	
%Less than $60 K	26.5
%$60–125 K	38.5
%$125 K or more	28.9
%Missing	6.1
Behavioral variables	
Time spent in cars (min/day)	48.7 (46.4)
Sitting for work (hr/day)^a^	3.07 (3.13)
TV viewing (hr/day)	1.70 (1.21)
Leisure-time computer use (min/day)	40.3 (59.0)
LTPA (min/day)	29.2 (48.9)
Energy intake (kJ/day)	7186 (2760)
Alcohol (g/day)	15.3 (18.9)
Medication use	
Medication for BP, %yes	16.1
Medication for cholesterol/triglycerides, %yes	11.6
Markers of cardio-metabolic risk	
BMI (kg/m^2^)	27.7 (5.2)
Waist circumference (cm)	93.3 (14.2)
Systolic BP (mmHg)	124.9 (17.0)
Diastolic BP (mmHg)	73.4 (10.6)
Triglycerides (mmol/L)^b^	1.10 (0.80, 1.60)
HDL-cholesterol (mmol/L)	1.54 (0.42)
Fasting plasma glucose (mmol/L)^b^	5.20 (4.90, 5.60)
2-h plasma glucose (mmol/L)^b^	5.30 (4.40, 6.40)
Clustered cardio-metabolic risk	− 0.05 (0.58)
Metabolic syndrome, %yes	26.5

a Mean (SD) of the whole sample.

b Data shown are median (25th, 75th percentile).

**Table 2:** Unstandardized regression coefficients and odds ratios (95%CI) for markers of cardio-metabolic risk according to the categories of time spent in cars, the 2011–12 AusDiab study.

Markers of cardio-metabolic risk		Time spent in cars		p for trend
	> 15 to ≤ 30 min/day (n = 749)	> 30 to ≤ 60 min/day (n = 851)	> 60 min/day (n = 673)	
BMI (kg/m^2^)	0.36 (− 0.22, 0.94)	0.56 (− 0.01, 1.12)	0.77 (0.16, 1.38)⁎	0.011
Waist circumference (cm)	0.75 (− 0.67, 2.16)	1.30 (− 0.08, 2.69)	1.50 (0.02, 2.98)⁎	0.034
Systolic BP (mmHg)^a^	0.69 (− 1.00, 2.37)	0.34 (− 1.32, 2.00)	1.60 (− 0.18, 3.37)	0.13
Diastolic BP (mmHg)^a^	0.56 (− 0.56, 1.68)	0.71 (− 0.39, 1.81)	0.69 (− 0.50, 1.87)	0.26
Triglycerides (log, mmol/L)^b^	0.016 (− 0.015, 0.047)	0.017 (− 0.013, 0.048)	0.002 (− 0.030, 0.035)	0.94
HDL-cholesterol (mmol/L)^b^	− 0.001 (− 0.042, 0.040)	− 0.019 (− 0.059, 0.021)	− 0.014 (− 0.057, 0.029)	0.36
Fasting plasma glucose (log, mmol/L)	0.005 (− 0.007, 0.017)	0.008 (− 0.004, 0.019)	0.013 (0.000, 0.026)⁎	0.044
2-h plasma glucose (log, mmol/L)	− 0.003 (− 0.036, 0.030)	− 0.022 (− 0.054, 0.011)	0.013 (− 0.021, 0.048)	0.72
Clustered cardio-metabolic risk^a^, ^b^	0.040 (− 0.022, 0.102)	0.062 (0.001, 0.123)⁎	0.076 (0.011, 0.141)⁎	0.017
Metabolic syndrome^c^	1.07 (0.82, 1.41)	1.13 (0.86, 1.47)	0.93 (0.70, 1.24)	0.70

Reference: ≤ 15 min/day (n = 527).

Analyses adjusted for age, gender, education, work status, marital status, having a child or children in the household, household income, sitting for work, TV viewing, leisure-time computer use, LTPA, energy intake, alcohol consumption, and corrected for clustering.

⁎ p < 0.05

a Further adjusted for medication use for BP.

b Further adjusted for medication use for cholesterol/triglycerides.

c Odds ratios of having the metabolic syndrome.

**Table 3:** Unstandardized regression coefficients (95%CI) for BMI according to the categories of time spent in cars: stratified by gender, the 2011–12 AusDiab study.

Gender		Time spent in cars		p for trend
	> 15 to ≤ 30 min/day	> 30 to ≤ 60 min/day	> 60 min/day	
Women	0.17 (− 0.64, 0.98)	0.10 (− 0.72, 0.92)	0.61 (− 0.30, 1.52)	0.25
Men	0.65 (− 0.15, 1.46)	1.17 (0.42, 1.92)⁎⁎	1.00 (0.23, 1.77)⁎	0.007

Reference: ≤ 15 min/day Analyses adjusted for age, education, work status, marital status, having a child or children in the household, household income, sitting for work, TV viewing, leisure-time computer use, LTPA, energy intake, alcohol consumption, and corrected for clustering.

⁎ p < 0.05.

⁎⁎ p < 0.01.
