# Supplementary material for: Adverse associations of car time with markers of cardio-metabolic risk
Source: Prev Med. 2016 Feb;83:26–30. doi: 10.1016/j.ypmed.2015.11.029 (PMC5405044; doi:10.1016/j.ypmed.2015.11.029)
Supplement: Supplementary file 2 — Supplementary tables. [file mmc2.docx]

**Supplementary Table 1**. Unstandardized regression coefficients and odds ratios (95%CI) for markers of cardio-metabolic risk according to the categories of time spent in cars, adjusting for base covariates, the 2011–12 AusDiab study

| Markers of  cardio-metabolic risk |  | Time spent in cars |  | p for trend |
| --- | --- | --- | --- | --- |
|  | >15 to ≤30 min/day  (n=749) | >30 to ≤60 min/day  (n=851) | >60 min/day  (n=673) |  |
| BMI (kg/m^2^) | 0.39 (-0.19, 0.98) | 0.54 (-0.03, 1.11) | 0.75 (0.14, 1.36)* | 0.016 |
| Waist circumference (cm) | 0.85 (-0.59, 2.28) | 1.27 (-0.13, 2.68) | 1.43 (-0.06, 2.93) | 0.052 |
| Systolic BP (mmHg)^a^ | 0.70 (-0.99, 2.38) | 0.27 (-1.38, 1.93) | 1.49 (-0.28, 3.27) | 0.17 |
| Diastolic BP (mmHg)^a^ | 0.62 (-0.51, 1.74) | 0.71 (-0.39, 1.82) | 0.69 (-0.49, 1.87) | 0.27 |
| Triglycerides (log, mmol/L)^b^ | 0.017 (-0.014, 0.049) | 0.019 (-0.012, 0.049) | 0.003 (-0.030, 0.035) | 0.92 |
| HDL-cholesterol (mmol/L)^b^ | -0.004 (-0.046, 0.037) | -0.021 (-0.062, 0.019) | -0.015 (-0.059, 0.028) | 0.34 |
| Fasting plasma glucose (log, mmol/L) | 0.006 (-0.006, 0.018) | 0.008 (-0.004, 0.020) | 0.013 (0.000, 0.026)* | 0.045 |
| 2-hr plasma glucose (log, mmol/L) | -0.002 (-0.035, 0.032) | -0.022 (-0.055, 0.011) | 0.012 (-0.023, 0.047) | 0.81 |
| Clustered cardio-metabolic risk^a,b^ | 0.046 (-0.017, 0.109) | 0.064 (0.002, 0.125)* | 0.077 (0.011, 0.143)* | 0.021 |
| Metabolic syndrome^c^ | 1.08 (0.83, 1.41) | 1.12 (0.86, 1.45) | 0.91 (0.69, 1.21) | 0.58 |

* *p* <0.05

Reference: ≤15 min/day (n=527)

Base covariates: age, gender, education, work status, marital status, having a child or children in the household, household income, energy intake, and alcohol consumption

Analyses corrected for clustering.

^a^ Further adjusted for medication use for BP

^b^ Further adjusted for medication use for cholesterol/triglycerides

^c^ Odds ratios of having the metabolic syndrome

**Supplementary Table 2**. Unstandardized regression coefficients and odds ratios (95%CI) for markers of cardio-metabolic risk according to the categories of time spent in cars, adjusting for base covariates and sitting for work, the 2011–12 AusDiab study

| Markers of  cardio-metabolic risk |  | Time spent in cars |  | p for trend |
| --- | --- | --- | --- | --- |
|  | >15 to ≤30 min/day  (n=749) | >30 to ≤60 min/day  (n=851) | >60 min/day  (n=673) |  |
| BMI (kg/m^2^) | 0.39 (-0.20, 0.97) | 0.52 (-0.05, 1.10) | 0.71 (0.10, 1.32)* | 0.023 |
| Waist circumference (cm) | 0.84 (-0.59, 2.27) | 1.24 (-0.17, 2.64) | 1.34 (-0.16, 2.84) | 0.070 |
| Systolic BP (mmHg)^a^ | 0.70 (-0.98, 2.39) | 0.32 (-1.34, 1.97) | 1.59 (-0.18, 3.36) | 0.14 |
| Diastolic BP (mmHg)^a^ | 0.62 (-0.51, 1.74) | 0.71 (-0.40, 1.81) | 0.68 (-0.50, 1.86) | 0.28 |
| Triglycerides (log, mmol/L)^b^ | 0.017 (-0.014, 0.049) | 0.018 (-0.013, 0.048) | 0.000 (-0.032, 0.033) | 0.97 |
| HDL-cholesterol (mmol/L)^b^ | -0.004 (-0.045, 0.037) | -0.020 (-0.060, 0.020) | -0.012 (-0.055, 0.031) | 0.42 |
| Fasting plasma glucose (log, mmol/L) | 0.006 (-0.006, 0.018) | 0.007 (-0.004, 0.019) | 0.013 (-0.000, 0.025) | 0.056 |
| 2-hr plasma glucose (log, mmol/L) | -0.002 (-0.035, 0.032) | -0.022 (-0.055, 0.011) | 0.012 (-0.023, 0.046) | 0.83 |
| Clustered cardio-metabolic risk^a,b^ | 0.046 (-0.017, 0.109) | 0.062 (0.000, 0.124)* | 0.072 (0.006, 0.138)* | 0.031 |
| Metabolic syndrome^c^ | 1.08 (0.83, 1.41) | 1.12 (0.86, 1.45) | 0.91 (0.69, 1.21) | 0.58 |

* *p* <0.05

Reference: ≤15 min/day (n=527)

Base covariates: age, gender, education, work status, marital status, having a child or children in the household, household income, energy intake, and alcohol consumption

Analyses corrected for clustering.

^a^ Further adjusted for medication use for BP

^b^ Further adjusted for medication use for cholesterol/triglycerides

^c^ Odds ratios of having the metabolic syndrome

**Supplementary Table 3**. Unstandardized regression coefficients and odds ratios (95%CI) for markers of cardio-metabolic risk according to the categories of time spent in cars, adjusting for base covariates and TV viewing, the 2011–12 AusDiab study

| Markers of  cardio-metabolic risk |  | Time spent in cars |  | p for trend |
| --- | --- | --- | --- | --- |
|  | >15 to ≤30 min/day  (n=749) | >30 to ≤60 min/day  (n=851) | >60 min/day  (n=673) |  |
| BMI (kg/m^2^) | 0.39 (-0.19, 0.98) | 0.58 (0.01, 1.16)* | 0.81 (0.19, 1.42)* | 0.008 |
| Waist circumference (cm) | 0.85 (-0.58, 2.28) | 1.41 (0.01, 2.81)* | 1.61 (0.12, 3.11)* | 0.025 |
| Systolic BP (mmHg)^a^ | 0.69 (-1.00, 2.37) | 0.31 (-1.34, 1.97) | 1.52 (-0.26, 3.29) | 0.16 |
| Diastolic BP (mmHg)^a^ | 0.62 (-0.51, 1.74) | 0.77 (-0.34, 1.87) | 0.75 (-0.43, 1.94) | 0.22 |
| Triglycerides (log, mmol/L)^b^ | 0.018 (-0.013, 0.049) | 0.020 (-0.011, 0.051) | 0.005 (-0.027, 0.038) | 0.78 |
| HDL-cholesterol (mmol/L)^b^ | -0.005 (-0.046, 0.036) | -0.024 (-0.064, 0.016) | -0.019 (-0.062, 0.024) | 0.25 |
| Fasting plasma glucose (log, mmol/L) | 0.006 (-0.006, 0.018) | 0.008 (-0.004, 0.020) | 0.014 (0.001, 0.027)* | 0.031 |
| 2-hr plasma glucose (log, mmol/L) | -0.001 (-0.034, 0.032) | -0.020 (-0.053, 0.013) | 0.015 (-0.020, 0.050) | 0.68 |
| Clustered cardio-metabolic risk^a,b^ | 0.047 (-0.016, 0.110) | 0.069 (0.008, 0.131)* | 0.085 (0.019, 0.150)* | 0.009 |
| Metabolic syndrome^c^ | 1.09 (0.83, 1.43) | 1.14 (0.88, 1.48) | 0.94 (0.70, 1.24) | 0.72 |

* *p* <0.05

Reference: ≤15 min/day (n=527)

Base covariates: age, gender, education, work status, marital status, having a child or children in the household, household income, energy intake, and alcohol consumption

Analyses corrected for clustering.

^a^ Further adjusted for medication use for BP

^b^ Further adjusted for medication use for cholesterol/triglycerides

^c^ Odds ratios of having the metabolic syndrome

**Supplementary Table 4**. Unstandardized regression coefficients and odds ratios (95%CI) for markers of cardio-metabolic risk according to the categories of time spent in cars, adjusting for base covariates and leisure-time computer use, the 2011–12 AusDiab study

| Markers of  cardio-metabolic risk |  | Time spent in cars |  | p for trend |
| --- | --- | --- | --- | --- |
|  | >15 to ≤30 min/day  (n=749) | >30 to ≤60 min/day  (n=851) | >60 min/day  (n=673) |  |
| BMI (kg/m^2^) | 0.40 (-0.18, 0.98) | 0.58 (0.01, 1.15)* | 0.80 (0.19, 1.41)** | 0.009 |
| Waist circumference (cm) | 0.87 (-0.55, 2.30) | 1.36 (-0.04, 2.76) | 1.55 (0.05, 3.04)* | 0.034 |
| Systolic BP (mmHg)^a^ | 0.69 (-1.00, 2.38) | 0.30 (-1.36, 1.95) | 1.49 (-0.28, 3.27) | 0.17 |
| Diastolic BP (mmHg)^a^ | 0.62 (-0.51, 1.74) | 0.73 (-0.37, 1.83) | 0.69 (-0.49, 1.88) | 0.27 |
| Triglycerides (log, mmol/L)^b^ | 0.019 (-0.013, 0.050) | 0.019 (-0.012, 0.049) | 0.004 (-0.028, 0.037) | 0.87 |
| HDL-cholesterol (mmol/L)^b^ | -0.006 (-0.047, 0.035) | -0.022 (-0.062, 0.018) | -0.018 (-0.061, 0.025) | 0.30 |
| Fasting plasma glucose (log, mmol/L) | 0.006 (-0.006, 0.018) | 0.008 (-0.004, 0.020) | 0.014 (0.001, 0.026)* | 0.040 |
| 2-hr plasma glucose (log, mmol/L) | -0.001 (-0.034, 0.033) | -0.021 (-0.054, 0.012) | 0.013 (-0.021, 0.048) | 0.75 |
| Clustered cardio-metabolic risk^a,b^ | 0.048 (-0.015, 0.111) | 0.066 (0.005, 0.128)* | 0.081 (0.015, 0.147)* | 0.015 |
| Metabolic syndrome^c^ | 1.10 (0.84, 1.43) | 1.12 (0.86, 1.46) | 0.93 (0.70, 1.24) | 0.66 |

* *p* <0.05

Reference: ≤15 min/day (n=527)

Base covariates: age, gender, education, work status, marital status, having a child or children in the household, household income, energy intake, and alcohol consumption

Analyses corrected for clustering.

^a^ Further adjusted for medication use for BP

^b^ Further adjusted for medication use for cholesterol/triglycerides

^c^ Odds ratios of having the metabolic syndrome

**Supplementary Table 5**. Unstandardized regression coefficients and odds ratios (95%CI) for markers of cardio-metabolic risk according to the categories of time spent in cars, adjusting for base covariates and LTPA, the 2011–12 AusDiab study

| Markers of  cardio-metabolic risk |  | Time spent in cars |  | p for trend |
| --- | --- | --- | --- | --- |
|  | >15 to ≤30 min/day  (n=749) | >30 to ≤60 min/day  (n=851) | >60 min/day  (n=673) |  |
| BMI (kg/m^2^) | 0.35 (-0.24, 0.93) | 0.50 (-0.08, 1.07) | 0.71 (0.10, 1.32)* | 0.022 |
| Waist circumference (cm) | 0.72 (-0.71, 2.14) | 1.15 (-0.25, 2.55) | 1.32 (-0.17, 2.81) | 0.068 |
| Systolic BP (mmHg)^a^ | 0.69 (-1.00, 2.37) | 0.27 (-1.39, 1.92) | 1.48 (-0.29, 3.25) | 0.17 |
| Diastolic BP (mmHg)^a^ | 0.56 (-0.56, 1.68) | 0.66 (-0.44, 1.76) | 0.64 (-0.54, 1.82) | 0.31 |
| Triglycerides (log, mmol/L)^b^ | 0.015 (-0.016, 0.046) | 0.016 (-0.014, 0.047) | 0.000 (-0.032, 0.033) | 0.97 |
| HDL-cholesterol (mmol/L)^b^ | 0.000 (-0.041, 0.041) | -0.017 (-0.057, 0.023) | -0.011 (-0.054, 0.031) | 0.42 |
| Fasting plasma glucose (log, mmol/L) | 0.005 (-0.007, 0.017) | 0.007 (-0.005, 0.019) | 0.012 (-0.000, 0.025) | 0.056 |
| 2-hr plasma glucose (log, mmol/L) | -0.004 (-0.037, 0.029) | -0.024 (-0.057, 0.008) | 0.009 (-0.025, 0.044) | 0.90 |
| Clustered cardio-metabolic risk^a,b^ | 0.038 (-0.024, 0.101) | 0.056 (-0.005, 0.117) | 0.070 (0.004, 0.134)* | 0.034 |
| Metabolic syndrome^c^ | 1.05 (0.81, 1.38) | 1.10 (0.84, 1.43) | 0.89 (0.67, 1.19) | 0.51 |

* *p* <0.05

Reference: ≤15 min/day (n=527)

Base covariates: age, gender, education, work status, marital status, having a child or children in the household, household income, energy intake, and alcohol consumption

Analyses corrected for clustering.

^a^ Further adjusted for medication use for BP

^b^ Further adjusted for medication use for cholesterol/triglycerides

^c^ Odds ratios of having the metabolic syndrome
